# Thermodynamics of weight loss diets

**DOI:** 10.1186/1743-7075-1-15

**Published:** 2004-12-08

**Authors:** Eugene J Fine, Richard D Feinman

**Affiliations:** 1 Department of Nuclear Medicine, Jacobi Medical Center, Bronx, NY, USA; 2 Department of Biochemistry, SUNY Downstate Medical Center, Brooklyn, NY 11203, USA; 3 Department of Biochemistry, SUNY Downstate Medical Center, Brooklyn, NY 11203, USA

## Abstract

**Background:**

It is commonly held that "a calorie is a calorie", i.e. that diets of equal caloric content will result in identical weight change independent of macronutrient composition, and appeal is frequently made to the laws of thermodynamics. We have previously shown that thermodynamics does not support such a view and that diets of different macronutrient content may be expected to induce different changes in body mass. Low carbohydrate diets in particular have claimed a "metabolic advantage" meaning more weight loss than in isocaloric diets of higher carbohydrate content. In this review, for pedagogic clarity, we reframe the theoretical discussion to directly link thermodynamic inefficiency to weight change. The problem in outline: Is metabolic advantage theoretically possible? If so, what biochemical mechanisms might plausibly explain it? Finally, what experimental evidence exists to determine whether it does or does not occur?

**Results:**

Reduced thermodynamic efficiency will result in increased weight loss. The laws of thermodynamics are silent on the existence of variable thermodynamic efficiency in metabolic processes. Therefore such variability is permitted and can be related to differences in weight lost. The existence of variable efficiency and metabolic advantage is therefore an empiric question rather than a theoretical one, confirmed by many experimental isocaloric studies, pending a properly performed meta-analysis. Mechanisms are as yet unknown, but plausible mechanisms at the metabolic level are proposed.

**Conclusions:**

Variable thermodynamic efficiency due to dietary manipulation is permitted by physical laws, is supported by much experimental data, and may be reasonably explained by plausible mechanisms.

## Background

Carbohydrate restriction as a general strategy for weight loss continues to gain in popularity and its utility and generally protective effect in lipid profile and glycemic control continues to be demonstrated, at least in an experimental setting [1-4]. The subject nonetheless remains controversial. Those critics who grant efficacy of low carbohydrate diets nonetheless contend that they act strictly by caloric restriction and there is no special effect of carbohydrate reduction. Beyond caloric restriction, several studies have shown increased weight loss on low carbohydrate diets compared to isocaloric low fat diets, the so-called metabolic advantage (see table 2). Although no clear experimental error has been demonstrated, critics continue to maintain that something must be wrong because the laws of thermodynamics would be violated [5], that "a calorie is a calorie" [6] We have previously shown [2,7] that this is not correct and it is our intention here to review the fundamental physics underlying the phenomenon of metabolic advantage. An outline may be described: Can metabolic advantage happen? If so, what mechanisms might account for such a phenomenon? Does it, in fact, occur?

**Table 2:** Isocaloric low carbohydrate (CHO) vs. higher carbohydrate investigations

***Reference***	*%CHO*	*%CHO*	*Wt. loss(kg) *± *SEM*		**p**
	Low	High	Low CHO arm (no. subjects)	High CHO arm	

Rabast et al (1978) [31]	10	68	**14.0 **± 1.4 (25)	9.8 ± 1.0 (20)	0.10
Rabast et al (1981) [32]	12	70	**12.5 **± 0.9 (7)	9.5 ± 0.7 (7)	<0.01
Golay, Allaz et al (1996) [33]	15	45	**8.9 **± 0.6 (22)	7.5 ± 0.5 (21)	0.1
Golay, Eigenheer et al (1996) [34]	25	45	**10.2 **± 0.7 (31)	8.6 ± 0.8 (37)	0.13
Piatti et al (1994) [35]	35	60	*4.5 *± *0.4 (10)*	***6.4 ***± *0.9 (15)*	0.3
Layman et al (2003) [36]	44	59	**7.5 **± 1.4 (12)	7.0 ± 1.4 (12)	0.8
Baba et al (1999) [38]	25	68	**8.3 **± 0.7 (7)	6.0 ± 0.6 (6)	<0.05
Lean et al (1997) [37]	35	58	**6.8 **± 0.8 (40)	5.6 ± 0.8 (42)	0.1
Young et al (1971) [39]	7	23	**16.2 **± 0.9 (3)	11.9 ± 0.8 (3)	<0.05
Greene et al (2003) [40]	5	55	**10.4 **± 2.1 (21)	7.7 ± 1.1 (21)	0.25

## Metabolic advantage: can it happen?

We have previously presented arguments that there is no violation of physical principles [2,7] and, ironically, that suggesting a change in body mass to be independent of macronutrient composition would itself be a violation of the second law of thermodynamics [7]. Here, we reframe these arguments in a more pedagogically direct way and we provide simple examples.

The misunderstanding that continues to be repeated in the expression "a calorie is a calorie" appears to be exclusive reference to the first law of thermodynamics. The difficulty with this theoretical approach is that it is only part of the relevant physics and its relationship to biologic systems. The first law says that in any transformation the total energy in the system can be accounted for by the heat added to the system, the work done by the system on its environment and the change in energy content of all the components of the system. It is important to understand, however, that the first law does not say what the relative distribution between these effects will be for any process. In fact, the first law does not even allow us to say whether the process will occur at all. To understand the progress of a physical change it is necessary to understand the second law which introduces an entity known as the entropy, S, a measure of disorder in all processes. In all real (irreversible) processes, entropy increases, usually written ΔS > 0. The most common marker of increasing entropy is heat, although it is by no means the only evidence for increased entropy.

In systems at constant temperature and pressure (i.e. biologic systems)), the first and second law are combined in the Gibbs Free Energy, ΔG, which represents the *maximum *useful work that can be performed by the process. The actual process however, in general derives less useful work than permitted by the theoretically available ΔG due to inefficiency in energy capture. A proper accounting of entropy and efficiency must be included if we are to understand energy utilization in biological and biochemical systems.

## Biological systems and thermodynamics

It is also important in the discussion of biological systems to understand that they are open systems, i.e. they take in nutrients and oxygen and excrete carbon dioxide, water, urea and other waste products, as well as heat. The importance with respect to weight considerations is that mass and energy are conserved (the more general statement of the first law of thermodynamics), but they are not conserved entirely within the organism.

**Figure 1:**
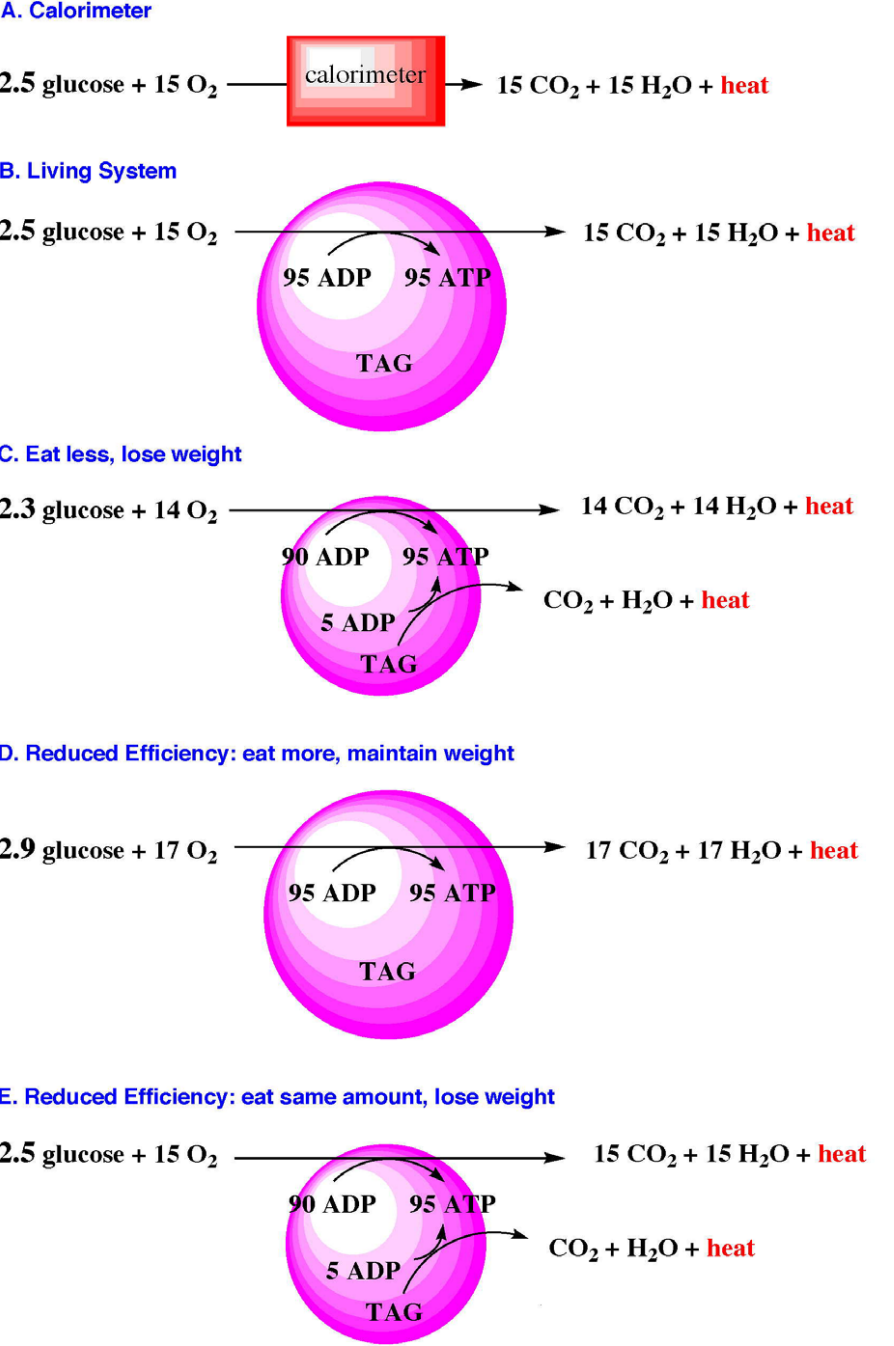
**A: **Oxidation of glucose in a calorimeter is completely inefficient. The products of oxidation are carbon dioxide and water, and all of the energy produced is released as heat. **1B: **To illustrate the proper interpretation of the first law of thermodynamics in living organisms we must consider that conservation of matter and energy includes excretion of products into the external environment. None of the products of oxidation (CO_2 _and H_2_O) remain within the organism. There is stoichiometric balance and no net weight change. Only the ATP, representing the useful energy, is retained. The wasted heat constitutes 60% of the energy of oxidation, while the efficiency is reflected in the retained ATP, available for reactions in the organism. Body fat stores are signified as TAG (triacylglycerol) **1C**. A common way of thinking of weight loss is from reduction of caloric intake. If our subject ingests 2.3 moles of glucose (or equivalent lipid and/or protein) and produces only 90 moles of ATP, then homeostasis will enlist body stores of fat (and/or lean body mass) to yield the additionally required 5 moles ATP. The additional resultant CO_2 _and H_2_O (and heat) will be excreted (and radiated) leading to weight loss. **1D**: If efficiency is reduced then our subject would have to eat more (e.g. 2.9 moles of glucose, or equivalent lipid/protein) to produce 95 moles of ATP and remain at the same weight. The additional CO_2 _and H_2_O produced will be excreted maintaining constant weight. **1E**: Under conditions of reduced metabolic efficiency (from 40% to about 38% in this example), 90 moles of ATP will be produced from oxidation of 2.5 moles glucose (or equivalent lipid/protein). The remaining 5 moles ATP needed for homeostasis must be made up from oxidation of body stores of lipid or lean mass. This results in weight loss, exactly as it does for the example of reduced caloric intake (Figure 1C).

To illustrate the proper interpretation of the first law of thermodynamics consider a subject whose resting energy expenditure is met by the production of 95 moles of ATP. Since oxidation of a single mole of glucose provides 38 moles of ATP, 2.5 moles of glucose will be needed to meet this individual's resting energy requirements. It is important to note that the resultant carbon dioxide, water, and heat are not retained within the organism. The useful retained energy is in the 95 moles of ATP (Figure 1B). (Similar equations could be written for lipid or protein but we restrict our discussion to glucose for simplicity).

The illustration above can be compared to the oxidation of glucose in a calorimeter in which no useful energy is obtained and the total energy of oxidation is measured as the heat produced. This process is completely inefficient. A traditional (Atwater) value for glucose obtained in the calorimeter is approximately 4 kilocalories of energy per gram (Figure 1A). By contrast, the living organism above metabolizes and oxidizes glucose so that approximately forty percent of the energy of oxidation is retained as useful ATP (38 moles per mole of glucose)) whereas sixty percent is released as heat, the inefficiency in this mode of oxidation. The entropy (i.e. the second law of thermodynamics) shows up in this *inefficiency*. The calorimeter heat can no longer be interpreted in a simple way. The energy stored in useful ATP represents the *efficiency *of 40% (neglecting the difference in entropy between the structures of the products and reactants). This value approximates the efficiency for oxidation of carbohydrate as well as lipid, whereas proteins are generally oxidized at a lower value of approximately 30–35% (Figure 1B).

### Summary of thermodynamics in living organism

1. The second law of thermodynamics dictates that there is an inevitable metabolic inefficiency in all biological and biochemical processes with heat and high entropy molecules (carbon dioxide, water, urea) as the most common products.

2. The first law of thermodynamics is satisfied in living (open) systems by properly accounting for the mass *excreted *and the heat radiated and exported in high entropy molecules.

### Weight loss due to reduced caloric intake

The most common example of weight loss is reduction of caloric intake. At the risk of oversimplification, if our subject ingests fewer than 2.5 moles of glucose and produces, for example, only 90 moles of ATP from food, then homeostasis would require enlisting endogenous body stores for further oxidation. This oxidation would then provide the additional 5 moles of ATP required. Oxidation of body stores (lipid or lean body mass) will result in production of additional carbon dioxide, urea, water and heat. The excretion of these products will result in weight loss. (Figure 1C).

### Weight loss due to increased metabolic inefficiency

The implication of the first and second laws of thermodynamics is that reduced efficiency has precisely the same result as reduced caloric intake. One conceptually simple means of reducing efficiency involves the process of *uncoupling *in mitochondria. ATP is produced in a variety of cellular locations. Glycolysis produces a net of two ATP's per molecule of glucose, in the cell cytoplasm. On the other hand, we recall that 36 additional molecules of ATP are produced from glucose as a result of the mitochondrial TCA cycle and electron transport. A critical part of the process involves the development of a hydrogen ion gradient across the mitochondrial membrane. This concentration gradient provides the energy that is converted into ATP as hydrogen ions pass down the gradient through the ATP synthase particle, entirely analogous to the energy in a high-pressure gas in a cylinder with a movable piston. (The expansion of the gas is like diffusion down a gradient: It does work against the piston). In the mitochondrion the energy of moving down the gradient is captured in ATP, the medium of exchange for the performance of work within cells. This capture of energy, referred to as *coupling *the energy to the formation of ATP, is the essential process permitting work to be done by living systems.

There are known endogenous and pharmacologic agents, which result in uncoupling the formation of ATP from the dissipation of the gradient. Uncouplers such as 2, 4-dinitrophenol bypass ATP synthase and cause hydrogen ion gradient dissipation without ATP formation that can result in organ dysfunction causing death. More modest degrees of uncoupling may be caused by the class of endogenous compounds we know as uncoupling proteins (UCP's). Three different isoforms, UCP1, UCP2 and UCP3 have been identified thus far in mammalian tissues. While the overall and relative physiologic importance of these proteins remains incompletely understood in human tissues, UCP1 has been shown in mice [8] to result in modest degrees of uncoupling in brown fat. Elevation of fatty acid concentration has been associated with induction of UCP3 and even with pathologic reductions of myocardial efficiency in rat heart [9]. For purposes of illustration, then, we may consider that there may be physiologic triggers that result in oxidative uncoupling, reducing the overall efficiency of glucose metabolism. For example if efficiency is reduced from 40% to 35%, the result will be the production of only 34 moles of ATP instead of the usual 38. While this represents a mechanism better demonstrated in rats than humans, our subject would require more glucose to make 95 moles of ATP. Now 2.9 moles of glucose would be required to produce 95 moles ATP. Our subject would either eat more and stay at the same weight (Figure 1D) or would eat 2.5 moles of glucose, the same amount as previously, but would produce less ATP. By eating only 2.5 moles of glucose our subject's metabolism would enlist oxidation of body stores to make up the additional ATP needed for homeostasis. This would result in weight loss exactly as it did for reduced caloric intake. (Figure 1D).

The essence of the second law of thermodynamics is that it *guarantees inefficiency in all metabolic processes*. However, variation of efficiency is not excluded. In fact, the laws of thermodynamics are *silent *on the existence of variable efficiency. If efficiency can vary (as in the example of oxidative uncoupling) then "a calorie is a calorie" is no longer a true statement. The role of uncoupling proteins in humans, as indicated, is as yet incompletely defined [10]. However, thermodynamic principles permit variable efficiency, and its existence must be determined empirically.

## Metabolic advantage: how could it happen?

It is possible that metabolic efficiency may be decreased by oxidative uncoupling as described above. Polymorphisms connecting uncoupling proteins with obesity or propensity to gain weight have been identified in humans [11,12] although these are not firmly established and the effect of dietary intervention is unknown. Other mechanisms are better understood and are described below.

### Substrate cycling and protein turnover

Substrate or "futile" cycles refer to the dynamic process that must accompany the thermodynamic steady state [13]. In particular, increased cycling of metabolic intermediates utilizes ATP and generates heat. The simplest examples are the numerous kinase-phosphatase pairs that regulate metabolism. In addition, although not generally considered in the category of substrate cycling, inefficiency results from the repeated breakdown and re-synthesis of proteins, lipids, and carbohydrates in cycles that use ATP for no apparent net gain. Such mechanisms, however, far from futile, allow for precision in the regulation of metabolism and constitute one of the uses of ATP. Protein turnover, in particular, provides for error correction or removal of "old" or damaged proteins. The effect of metabolic path on the energetics of oxidation is illustrated in Table 1 which summarizes the analysis from our earlier paper [2]. In this example, a mole of glucose directly oxidized to CO_2 _and water generates 38 moles of ATP with an overall efficiency of about 38.5%. On the other hand, if glucose is first incorporated into glycogen, followed by hydrolysis of the glucose and subsequent oxidation, 2 moles of ATP are lost per mole in this cycle with overall efficiency reduced to 35%. Similarly an amino acid from an "average" protein, when directly oxidized to CO_2_, produces ATP with an efficiency of about 33%. If the amino acid is first incorporated into a protein and later hydrolyzed and oxidized, four ATP's per molecule are used for synthesis of the peptide bond. This reduces the efficiency to 27%. Smaller degrees of inefficiency are seen for lipid cycles (Table 1) but multiple cycles may have a cumulative effect. It is estimated, for example, that half of depot fatty acids in triacylglycerol have been through at least one cycle [14]. It should be apparent that variation in efficiency is not a thermodynamic issue but an empiric question to be determined by the requirements of metabolism.

**Table 1:** Effect of Path on energetics of oxidation

**Macronutrient & path**	**Mass**	**ATP/mole**	**Kcal/gm**	**Efficiency (%)**
Glucose → CO_2_	180	38	1.54	38.5
Glucose → glycogen → glucose → CO_2_	180	36	1.40	35
"Average" AA → CO_2_			1.32	33
AA → Protein → AA → CO_2_		-4	1.08	27
Palmitate → CO_2_	256	129	3.68	40.9
Palmitate → Ketone → CO_2_	256	121	3.45	38.3

*Adapted from Feinman, Fine: 2003 Metabolic Syndrome and Related Disorders (1): 209–219 [2]

### Thyrotoxicosis

Thyroid hormone decreases efficiency possibly by mechanisms involving both uncoupling and cycling described above: oxidative uncoupling as well as increased futile cycling of intermediates [15]. It is observed in thyrotoxic mice that UCP1 decreases efficiency in brown fat at the mitochondrial level [8]. In humans, the role of UCP1 in thyrotoxicosis is less certain due to the relative paucity of brown fat. On the other hand, activation of the adrenergic system via phosphoenolpyruvate carboxykinase ultimately increases "futile" metabolic cycling of intermediates ([15]). Thyrotoxicosis is well known to result in weight loss, often with increased food intake and increased generation of heat, indicative of metabolic inefficiency. The use of thyroid hormone has even been suggested therapeutically to induce weight loss in obese individuals, although its toxicity has limited this application. Inefficiency in metabolic processes with weight loss and increased heat generation, therefore, is known to occur on clinical grounds. Even without a complete understanding of the relative importance of different underlying cellular mechanisms in humans, the *potential for biochemical processes to reduce their efficiency must be considered established as a feature of mammalian metabolism*.

### Protein induced protein turnover

There is abundant evidence that dietary protein stimulates protein breakdown and re-synthesis. In particular, branched chain amino acids, and especially leucine, are documented to act as nutritional signals acting via both the insulin and mTOR signaling pathways [16-18]. On the macroscopic level, the energetic cost of protein turnover is demonstrable as excess heat generated during a high protein meal. Thermogenesis (thermogenic effect of feeding; old name: specific dynamic action) has been defined as the extra heat generated during a meal due to digestion or metabolism. Johnston et al [19] compared the energy expended during 9 hour intravenous feedings of a high protein meal, vs. an isocaloric high carbohydrate meal; both contrasted with a 9 hour fast. The protein meal, with 70% of its caloric value due to protein, had significantly greater thermogenesis than the high carbohydrate meal (70% of calories from carbohydrate). These data have been reproduced in numerous studies [19-22]. The overall energy costs of protein turnover and synthesis have been estimated in various animal species, including man, and tabulated by Vernon Young ([23]), based on data from other investigators [24-26]. Despite the substantial experimental difficulties involved, the cost of protein synthesis clusters at around 4–5 kcal/gram in 8 species of birds, marsupials and mammals, including man. The high energetic cost is understandable in view of the multiple ATP-requiring processes involved. The cost of protein turnover can reduce efficiency from 33% to 27%, merely in the formation and hydrolysis of a single peptide bond (requiring 4 ATP's per bond formed: Table 1). In addition, protein processes that are ATP-dependent include formation of the ribosomal initiation complex, translation and folding of the protein, and protein degradation (both ubiquitin-dependent and -independent pathways) [23]. The energy costs of protein turnover could therefore account for a metabolic advantage in high protein diets, independent of carbohydrate content. This mechanism may also contribute to inefficiency in low carbohydrate diets, often high in protein.

### Gluconeogenesis-stimulated protein turnover in carbohydrate restriction

The following hypothesis is suggested from classic studies of starvation done in chronically fasted obese individuals [27,28]. The brain's metabolism requires 100 grams of glucose per day. In the early phase of starvation, glycogen stores are rapidly reduced, so the requirement for glucose, is met by gluconeogenesis. Approximately 15–20 grams are available from glycerol production due to lipolysis, but fatty acid oxidation generally cannot be used to produce glucose. Therefore, protein breakdown must supply the rest of substrate for conversion to glucose in the early phases of starvation. By 6 weeks of starvation, ketone bodies plus glycerol can replace 85% of the brain's metabolic needs, the remainder still arising from gluconeogenesis due to protein. It should be mentioned that, since the fundamental role of ketones is to spare protein, it might be expected that the reliance on protein would actually decrease with time, perhaps relating to the anecdotal observation of "hitting the wall" on weight loss diets.

Very low carbohydrate diets, in their early phases, also must supply substantial glucose to the brain from gluconeogenesis. For example, the early phase of the popular Atkins or Protein Power diet restricts dieters to about 20–30 grams of carbohydrate per day, leaving 60–65 grams to be made up from protein-originated gluconeogenesis. One hundred grams of an "average" protein can supply about 57 grams of glucose so 110 grams protein would be needed to provide 60–65 grams glucose. Increased gluconeogenesis has been directly confirmed using tracer studies on day 11 of a very low carbohydrate diet (approx 8 grams/day) [29]. If indeed, 110 grams of endogenous protein is broken down for gluconeogenesis and re-synthesized, the energy cost, at 4–5 kcal/gram could amount to as much as 400–600 kcal/day. This is a sizable metabolic advantage. Of course, the source of protein for gluconeogenesis may be dietary rather than endogenous. Whereas endogenous protein breakdown is likely to evoke energetically costly re-synthesis in an organism in homeostasis, dietary protein may conserve energy. The source of protein for the observed gluconeogenesis [29] remains an open question, but there is no a priori reason to exclude endogenous rather than dietary sources. This is therefore a hypothesis that would need to be tested. The extent to which the protein for gluconeogenesis is supplied by endogenous protein would explain very high-energy costs. It should be noted, however, that even if limited to breakdown of dietary protein sources, there would be some energy cost associated with gluconeogenesis.

## Metabolic advantage: does it happen?

Having established that there is no theoretical barrier to metabolic advantage and that there are plausible mechanisms that could account for such an effect, we must ask whether it can be demonstrated experimentally, that is, whether the proposed effects are of sufficient magnitude to be a practical feature of weight reduction strategies, in particular very low carbohydrate diets. If so there will be increased weight loss for the same caloric intake, or metabolic advantage. A recent animal model provides support for greater metabolic inefficiency in rats fed carbohydrate restricted diets compared with higher carbohydrate, leading to excess weight loss [30]. Human data in Table 2 illustrates 10 clinical trials of isocaloric diets with a lower versus higher carbohydrate arm in each trial [31-40]. It can be seen that the lower carbohydrate arm in 9 of 10 studies demonstrates increased weight reduction in comparison with the higher carbohydrate arm. Three of the studies show statistical significance (p < 0.05 or better). Even without statistical significance of individual studies, however, the likelihood that the lower carbohydrate arm would have an advantage in 9 of 10 studies is equivalent to the likelihood of 9 coin toss experiments having excess heads in comparison to excess tails. The 9^th ^binomial coefficient shows this probability to be p < 0.01.

While the above suggests the possibility of metabolic advantage, it does not prove it, nor do we know the magnitude of the effect, or the factors that control it. The studies above were chosen from among those quoted by many of the authors who have disputed the existence of metabolic advantage. Nonetheless, a formal meta-analysis would be necessary to avoid the possibility of conscious or unconscious bias in their selection. Further, it would be necessary to establish evidence that energetically costly metabolic processes are more prevalent in low carbohydrate diets than in diets of higher carbohydrate content. Whereas the proposed mechanisms are plausible, they need to be proven.

## Conclusions

Thermodynamics is not the limiting factor behind the concept of metabolic advantage. On the contrary, thermodynamics *guarantees *inefficiency in all metabolic processes and is silent on the possibility that inefficiency may be augmented in some instances. A familiar example of inefficiency is thyrotoxicosis, with attendant weight loss and heat generation despite unchanged or increased caloric consumption. The theoretical possibility of inefficiency and metabolic advantage due to macronutrient compositional change exists, but demonstration of the phenomenon can only be resolved experimentally. Isocaloric dietary studies with a low vs. a higher carbohydrate arm support the experimental possibility of metabolic advantage. A formal meta-analysis would be required to evaluate this more objectively. Further studies, including tracer methods, would be required to establish mechanisms. The presence of high quantities of dietary protein (often a feature of low carbohydrate diets) is known to stimulate protein turnover, an energetically costly process. However, it is unclear whether this is the only factor, or whether it is necessary for metabolic advantage to occur. In particular, obligate gluconeogenesis from endogenous sources may also contribute to induction of protein turnover.

## Competing interests

The author(s) declare that they have no competing interests.
